# Ocular surface disease index in Graves’ orbitopathy: a cross-sectional study

**DOI:** 10.3389/fendo.2024.1428185

**Published:** 2024-12-05

**Authors:** Maria Novella Maglionico, Giulia Lanzolla, Michele Figus, Giada Cosentino, Simone Comi, Michele Marinò, Ferruccio Santini, Chiara Posarelli

**Affiliations:** ^1^ Department of Surgical, Medical and Molecular Pathology, Ophthalmopathy Unit I, University of Pisa and University Hospital of Pisa, Pisa, Italy; ^2^ Department of Clinical and Experimental Medicine, Endocrinology Unit I-II, University of Pisa and University Hospital of Pisa, Pisa, Italy

**Keywords:** Graves’ orbitopathy, Graves’ disease, dry eye disease, thyroid autoimmunity, ocular surface disease, ocular surface disease index

## Abstract

**Introduction:**

Graves' Orbitopathy (GO) is an autoimmune disorder characterized by inflammation of orbital tissues, leading to various ocular manifestations, including ocular surface disease. This cross-sectional study aimed to assess the presence of ocular surface disease using the Ocular Surface Disease Index (OSDI) in patients with Graves' disease (GD) and moderate-to-severe active GO compared to those with GD and mild non-active GO. Additionally, we aimed to investigate the correlation between ocular surface disease and the eye features of GO.

**Methods:**

Consecutive GD patients with GO referred to the Ophthalmology and Endocrinology Units of the University Hospital of Pisa between June 2022 and February 2023 were enrolled. OSDI scores were obtained from 79 GD patients, categorized into moderate-to-severe active GO and mild non-active GO groups.

**Results:**

OSDI scores were significantly higher in patients with moderate-to-severe active GO compared to those with mild non-active GO (P=0.0006). A cutoff value of 33 for positive tests revealed a higher frequency of pathological OSDI in moderate-to-severe active GO patients compared to mild non-active GO patients (P=0.0221; OR 3.673, CI 1.277-9.531). Within the moderate-to-severe active GO group, a significant positive correlation was found between OSDI and Clinical Activity Score (CAS) (R= 0.3867, 95% CI from 0.1403 to 0.5880; P=0.0030). Using a cutoff value of 55 (the 75th percentile of the study population), patients with CAS ≥ 3 had a significantly higher proportion of pathological OSDI compared to those with CAS <3 (P=0.0039; OR 4.075, CI 1.619-10.39). Proptosis values ≥ 22 mm and the presence of lagophthalmos were identified as significant risk factors for ocular surface disease development (P=0.0406 and P=0.0493, respectively).

**Discussion:**

Our study highlights a significantly higher prevalence of ocular surface disease, as measured by OSDI, in patients with moderate-to-severe active GO compared to those with mild non-active disease. The degree of GO activity positively correlates with ocular surface involvement, and proptosis and lagophthalmos increase the risk of its occurrence. These findings emphasise the importance of assessing and managing ocular surface health in GO patients. Early identification and appropriate treatment of ocular surface disease need to be pursued to improve patient management.

## Introduction

Graves’ orbitopathy (GO) is an autoimmune disease affecting orbital fibro-adipose tissue due to the interplay between cellular and humoral immunity against thyrotropic hormone (TSH) receptor (TSH-R) and possibly other autoantigens shared by thyroid epithelial cells and orbital fibroblasts (1, 2). Clinical manifestations of GO can be ascribed to three features: inflammation of soft tissues, glycosaminoglycans overproduction, and an increase in adipose tissue (3, 4). Along with inflammation, proptosis and diplopia, ocular surface damage may occur, frequently preceding the classic clinical onset of GO (5–12). One of the leading causes of ocular surface damage in GO is dry eye syndrome (DED) which is increasingly observed in patients with autoimmune diseases, including thyroid disorders (8–10). Previous studies have reported the prevalence of DED in GO patients to vary between 65 and 95% (5, 6, 11).

Dry eye disease (DED) is a multifactorial disease of the ocular surface characterized by loss of tear film homeostasis accompanied by ocular symptoms. Tear film instability, hyperosmolarity, ocular inflammation and neurosensory abnormalities are the main trigger factors (8, 13). Ocular surface changes can lead to blurred vision, burning, itchiness, redness, grittiness in the eye, and photophobia, eventually contributing to the clinical manifestations of GO (8–10).

While the association between GO and DED is well-acknowledged, the precise mechanism underlying this relationship remains incompletely understood. Proptosis and lid alterations are believed to disrupt ocular surface homeostasis, leading to corneal exposure and increased tear evaporation due to tear film instability (14). Moreover, ocular surface tissues themselves may serve as direct targets for autoantibodies and inflammation originating in the retrobulbar space (15). Interestingly, the potential correlation between DED and the severity and activity of GO has never been investigated.

## Methods

### Study design

The study was aimed at evaluating the presence of ocular surface disease in patients with Graves’ disease (GD) and moderate-to-severe active GO compared with patients with GD and mild non-active GO by using the Ocular Surface Disease Index (OSDI) in a cross-sectional design, which entailed the inclusion of consecutive patients over 9 consecutive months. In addition, we aimed to investigate the correlation between ocular surface disease and the eye features of GO. Given the absence of pre-existing data on the topic, a formal sample size calculation was not performed before starting the study. However, a *post hoc* analysis was conducted to determine the sample size needed for sufficient power. The study was performed according to the institutional guidelines and with the International Conference on Harmonization Good Clinical Practice guidelines and the principles of the Declaration of Helsinki (1975) and its revised version of 2013. The study was approved by the local Ethic Committee “Area Vasta Nord-Ovest”, (approval no.24940). Data were anonymized for collection and analysis, avoiding a privacy data breach.

### Setting

The investigation was carried out in a tertiary referral center from June 2022 to February 2023. MNM and GL collected the data and recorded them in a database. Database validation procedures included: allowed character checks, batch totals, missing records check, cardinality check, digits check, consistency check, control totals, cross-system consistency check, data type check, hash totals, limit check, logic check, presence check, range check, spelling and grammar check, and uniqueness check.

### Participants

Inclusion criteria were: 1) diagnosis of GD, namely a history of hyperthyroidism along with previous or present detectable serum thyrotropin receptor binding antibodies (TRAbs); 2) diagnosis of GO, according to the European Group on Graves’ Orbitopathy (EUGOGO) guidelines (4); 3) written, signed informed consent to data use and sample collection. Exclusion criteria were: 1) age < 18 years; 2) presence of corneal dystrophies; 3) local and/or systemic therapy with drugs known to have ocular toxicity; 4) presence of ocular or systemic pathologies that may interfere with assessments (e.g. diabetes mellitus, Sjogren’s syndrome); 5) previous decompressive orbitotomy or eye surgery related and/or unrelated to GO; 6) radioiodine treatment and/or external orbital radiotherapy in the last 12 months; 7) treatment with glucocorticoids or any other immune suppressive medication in the last three months. A total of 79 consecutive patients who satisfied the inclusion criteria and evaded the exclusion criteria were enrolled.

GO activity was assessed by Clinical Activity Score (CAS). GO severity was evaluated according to the EUGOGO criteria, which include the evaluation of eyelid retraction, proptosis, corneal involvement and the degree of diplopia (4).

### Outcomes

The primary outcome was to assess the presence of ocular surface disease by using OSDI in patients with moderate-to-severe active GO compared to patients with mild non-active GO. The secondary endpoints were: 1) the correlation between OSDI and CAS; 2) the correlation between OSDI and the eye features of GO namely proptosis, diplopia, eyelid aperture and lagophthalmos.

### Sources of data and measurements

An ophthalmological evaluation was performed in all patients, including 1) Hertel’s exophthalmometry (Handaya, Tokyo, Japan); 2) eyelid aperture; 3) CAS; 4) assessment of diplopia evaluated using a Gorman score (16) (patients were divided into four categories accordingly, as shown in **Table 1**); 5) assessment of the corneal status; 6) presence of lagophthalmos; 7) examination of the fundi; and 8) assessment of visual acuity, measured as best corrected visual acuity (BCVA) in Logarithm of the Minimum Angle of Resolution (LogMar). Symptoms such as photophobia, lacrimation, irritation, blurred vision, burning, itchiness or grittiness and dryness were also investigated. Patients were asked to complete the OSDI questionnaire (**Table 2**). Developed by the Outcome Research Group at Allergan (Irvine, Calif) and copyrighted by AbbVie Inc. (North Chicago, Illinois, USA), this questionnaire is widely used to assess several aspects of ocular surface disease (17).

**Table 1:** Demographic and clinical features of patients with Graves’ orbitopathy (GO) based on the severity and the activity of the disease.

	Moderate-to-severe active GO (n=57)	Mild non active GO (n=22)	Statistics
Gender	Males: 15 (26.31) Females: 42 (73.68)	Males: 4 (18.18) Females: 18 (81.81)	OR: 1.60 95 % CI from 0.49 to 4.91 P=0.56*
Age (years)	55.34 (10.30)	46.89 (11.51)	Mean difference: -8.45 95 % CI from -13.79 to -3.11 P=0.0023**
Smoking	Never smokers: 75 (47.4) Ex-smokers: 23 (14.5) Current smokers: 56 (35.4)	Never smokers: 63 (58.8) Ex-smokers: 10 (9.3) Current smokers: 30 (28)	Chi-squared test: 1.95 P=0.37***
Time since diagnosis of hyperthyroidism (months)	26 (14-81)	33 (20-75)	Mann-Whitney U: 424.5 P=0.56 †
Time since diagnosis of Graves’orbitopathy (months)	21 (12-49)	24 (12-34)	Mann-Whitney U: 461.5 P=0.85†
TSH (mU/L) NV: 0.4-4	1.67 (0.41-3.96)	0.99 (0.40-2.75)	Mann-Withney U: 462 P=0.52†
FT3 (ng/L) NV: 2.7-5.7	3.73 (1.001)	3.58 (1.14)	Mean difference: -0.15 95 % CI from -0.73 to -0.42 P=0.59**
FT4 (ng/dL) NV:0.70-1.70	1.18 (0.34)	1.15 (0.30)	Mean difference: -0.02 95 % CI from -0.21 to -0.15 P=0.76**
TRAbs (UI/L) NV: <1.5	6.53 (3.40-19.97)	1.96 (0.64-9.22)	Mann-Withney U: 154 P=0.08†
Proptosis	22.5 (18-24.5)	16 (14-21)	Mann-Withney U: 7.9 P=0.69†
Clinical activity score	4 (3-5)	1(0-2)	Mann-Withney U: 0 P<0.0001†
Eyelid aperture (mm)	13.59 (2.86)	10.77 (2.89)	Mean difference: -2.81 95 % CI from -4.25 to -1.37 P=0.0002**
Diplopia	Absent: 20 (35.02) Intermittent: 11 (19.2) Inconstant: 13 (22.8) Constant 13 (22.8)	Absent: 20 (90.9) Intermittent: 2 (9.1) Inconstant: 0 (0) Constant 0 (0)	Chi-squared test: 20.81 P=0.0001***
Visual acuity (decimals)	0.98 (0.09)	0.98 (0.09)	Mean difference: -0.24 95 % CI from -0.24 to -0.62 P=0.69**

Data are n (%), mean (SD) or median (IQR).

NV, normal values; TSH, thyrotropic hormone; FT3, free triiodothyronine; FT4, free thyroxine; TRAbs: anti-TSH receptor autoantibodies.

Statistical tests: *Fisher exact test; **Student’s t-test; ***Chi-squared test; †Mann-Whitney.

**Table 2:** Ocular Surface Disease Index (OSDI) Questionnaire.

Question	Response Options
*Category I (eye irritation symptoms)*	
1. Eyes sensitive to light	☐ Always ☐ Almost always ☐ Half of the time ☐ Sometimes ☐ Never
2. Gritty feeling in the eyes	☐ Always ☐ Almost always ☐ Half of the time ☐ Sometimes ☐ Never
3. Eye pain or irritation	☐ Always ☐ Almost always ☐ Half of the time ☐ Sometimes ☐ Never
4. Blurred vision	☐ Always ☐ Almost always ☐ Half of the time ☐ Sometimes ☐ Never
5. Poor vision	☐ Always ☐ Almost always ☐ Half of the time ☐ Sometimes ☐ Never
*Category II (fuctional disease)*	
6. Reading	☐ Always ☐ Almost always ☐ Half of the time ☐ Sometimes ☐ Never ☐ No response
7. Night driving	☐ Always ☐ Almost always ☐ Half of the time ☐ Sometimes ☐ Never ☐ No response
8. Working on a computer	☐ Always ☐ Almost always ☐ Half of the time ☐ Sometimes ☐ Never ☐ No response
9. Watching TV	☐ Always ☐ Almost always ☐ Half of the time ☐ Sometimes ☐ Never ☐ No response
*Category III (environmental triggers)*	
10. Windy conditions	☐ Always ☐ Almost always ☐ Half of the time ☐ Sometimes ☐ Never ☐ No response
11. Very dry places	☐ Always ☐ Almost always ☐ Half of the time ☐ Sometimes ☐ Never ☐ No response
12. Places with air conditioning	☐ Always ☐ Almost always ☐ Half of the time ☐ Sometimes ☐ Never ☐ No response

Patients were asked to mark the box that best represents their condition.

The OSDI is a validated tool for measuring the severity of DED, approved by the Food and Drugs Administration (FDA) for clinical trials (17). The questionnaire consists of 12 items, divided into three categories: symptoms of eye irritation, functional problems, and environmental triggers contributing to or causing ocular surface pathology. Each element is assigned a score from 0 to 4 and the total score ranges from 0 to 100. The score obtained can fall into four categories: 0-12: normal ocular surface; 13-22: mild dry eye condition; 23-32: moderate dry eye condition; 33-100: severe dry eye 16. In our study, results above 33 were considered pathological (8, 10, 11).

Demographic data, including age, gender, smoking habit, GD and GO duration, previous treatment for both GD and GO, and general clinical data were collected. The following blood tests were performed in all subjects: free thyroxine (FT4) (Vitros Immunodiagnostics, Raritan, NJ); free triiodothyronine (FT3) (Vitros Immunodiagnostics, Raritan, NJ); TSH (Immulite 2000, Siemens Healthcare, Gwynedd, UK); TRAbs (Brahms, Berlin, Germany).

### Statistical analysis

Continuous data are presented as mean and standard deviation (SD) or median (interquartile range IQR). The Shapiro-Wilk test for normality of distribution was performed on all variables. When appropriate, the following tests were performed: 1) Linear regression; 2) Mann-Whitney U; 3) Fisher exact test; 4) Chi-squared Test; 5) Student’s t-test; 6) Multiple Linear regression. Statistical analysis was performed by using GraphPad Prism, version 9.4.1. P-values < 0.05 were considered statistically significant.

## Results

**Figure 1:**
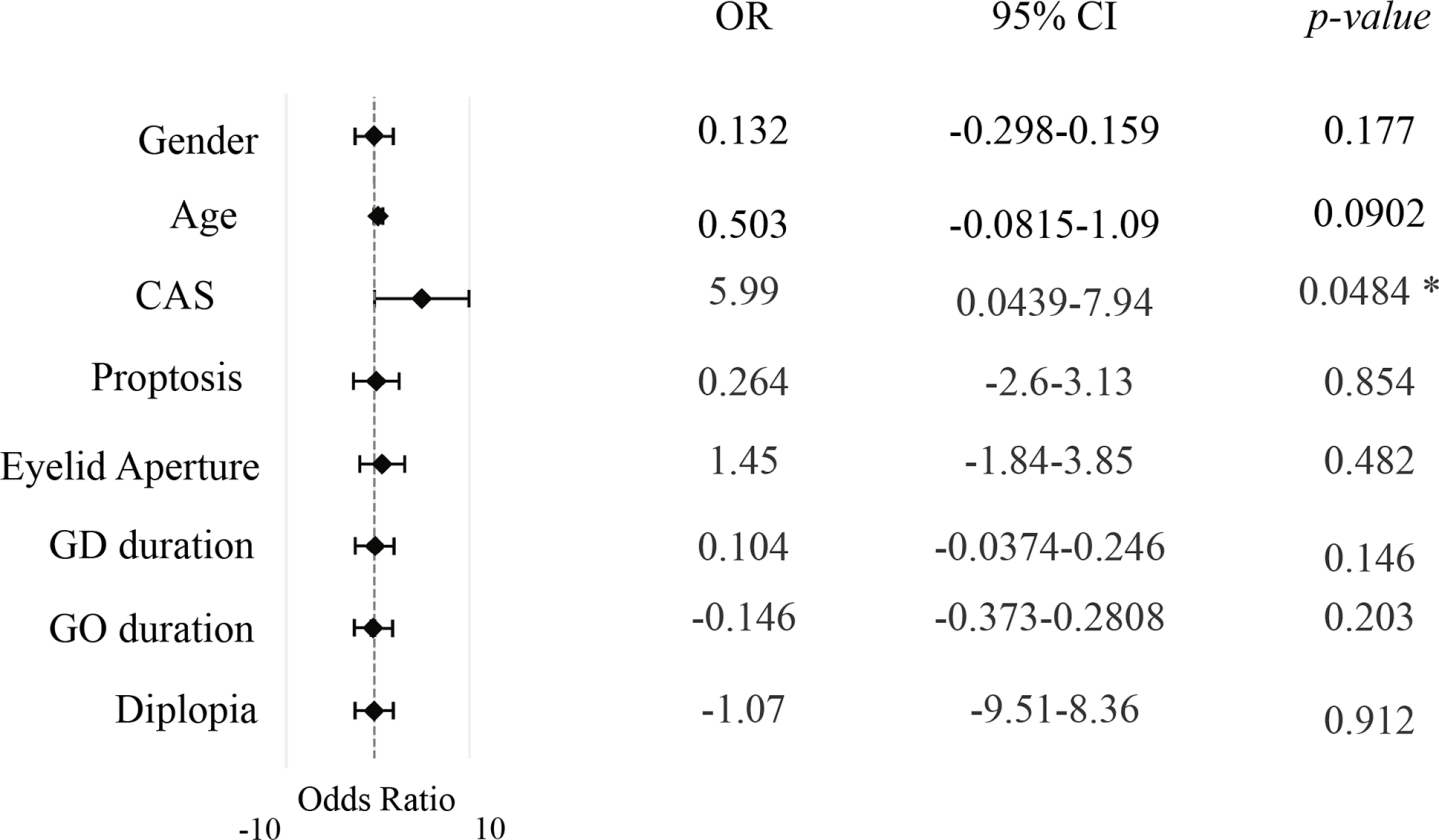
Multivariate effects of clinical and demographic key variables on Ocular Surface Disease Index (OSDI) in the cohort study population. The independent effects of age, CAS, proptosis, eyelid aperture, GD duration, GO duration and diplopia on the Ocular Surface Disease Index (OSDI) scores in the cohort. The analysis controls for potential confounders and reveals the unique contribution of each variable to the observed outcomes. P value significance: P <0.05, *. CAS, clinical activity score; GD, Graves’ Disease; GO, Graves’ Orbitopathy.

The OSDI was assessed in 79 patients, whose demographical and clinical data are reported in **Table 1**. The study population included 57 patients with moderate-to-severe active GO and 22 patients with mild non-active GO. The patient’s group did not differ in gender distribution, smoking habit, GD duration, and GO duration, while age was significantly different (P=0.0023). Notably, there was no correlation between age and OSDI (**Figure 1**). Sixty-five patients were on methimazole for hyperthyroidism treatment, but a minority of them (14 patients) had been previously treated with I^131^ (more than 12 months before enrolment) and were on levothyroxine (LT4) treatment for hypothyroidism. All patients were euthyroid. Among patients with moderate-to-severe active GO, 26 have been previously treated with ivGCs (more than 3 months before enrolling).

**Figure 2:**
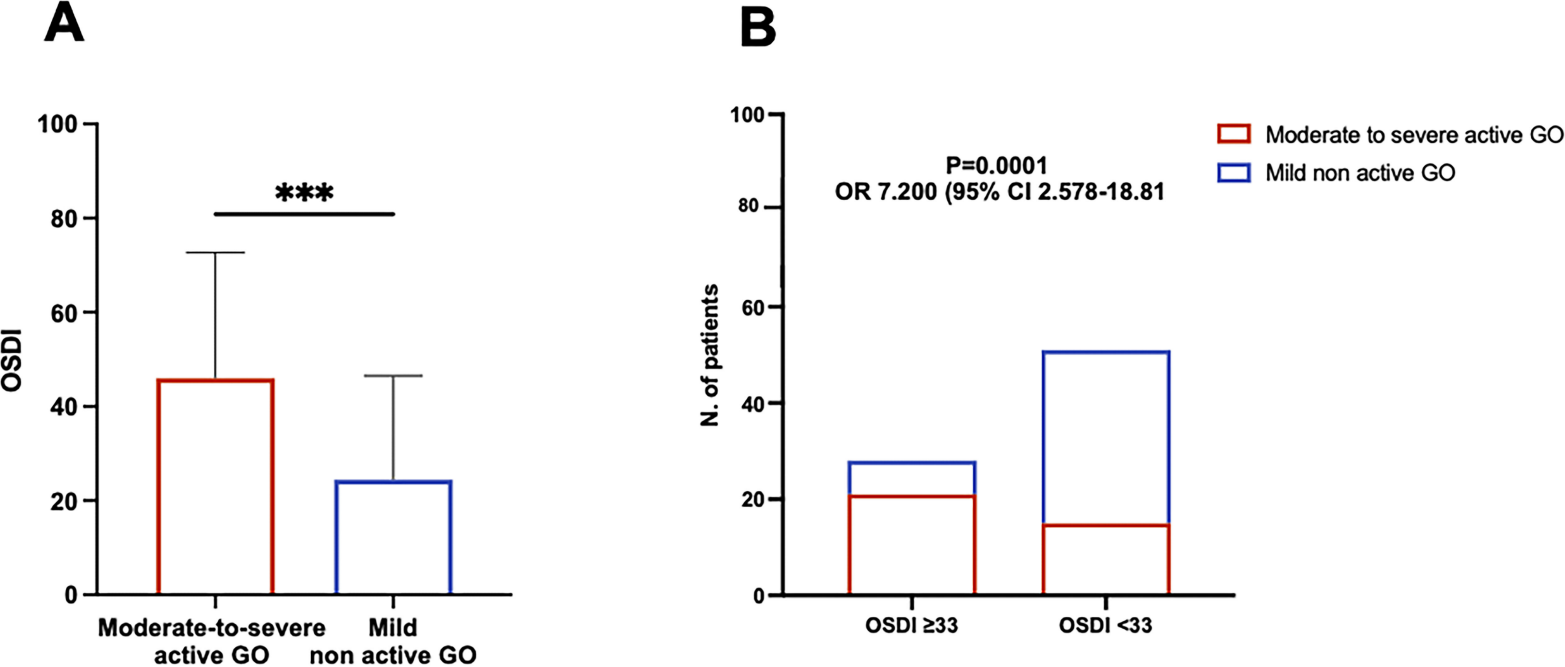
Patients with moderate-to-severe active Graves Orbitopathy (GO) experience worsened Ocular Surface Disease Index (OSDI) compared with mild non-active GO patients. **(A)** Comparison of the OSDI between patients with moderate-to-severe active GO and patients with mild non-active GO. The statistical significance was assessed by Mann-Whitney test. P value significance: P <0.001, ***. **(B)** Comparison of the frequency of pathological OSDI (>33) between patients with moderate-to-severe active GO and patients with mild non active GO. Statistical analysis was performed using Fisher exact test yielding both P value and odds ratio (OR).

The OSDI was significantly higher in patients with moderate-to-severe active GO compared with patients with mild non-active GO (P=0.0006 by Mann-Whitney; Mann-Whitney U: 318.5) (**Figure 2A**). Having established a cut-off value of 33 for positive tests, pathological OSDI was more frequent in patients with moderate to severe active GO than in those with mild non-active GO (P=0.0001; OR 7.200, 95% CI 2.578-18.81 by Fisher exact test) (**Figure 2B**).

**Figure 3:**
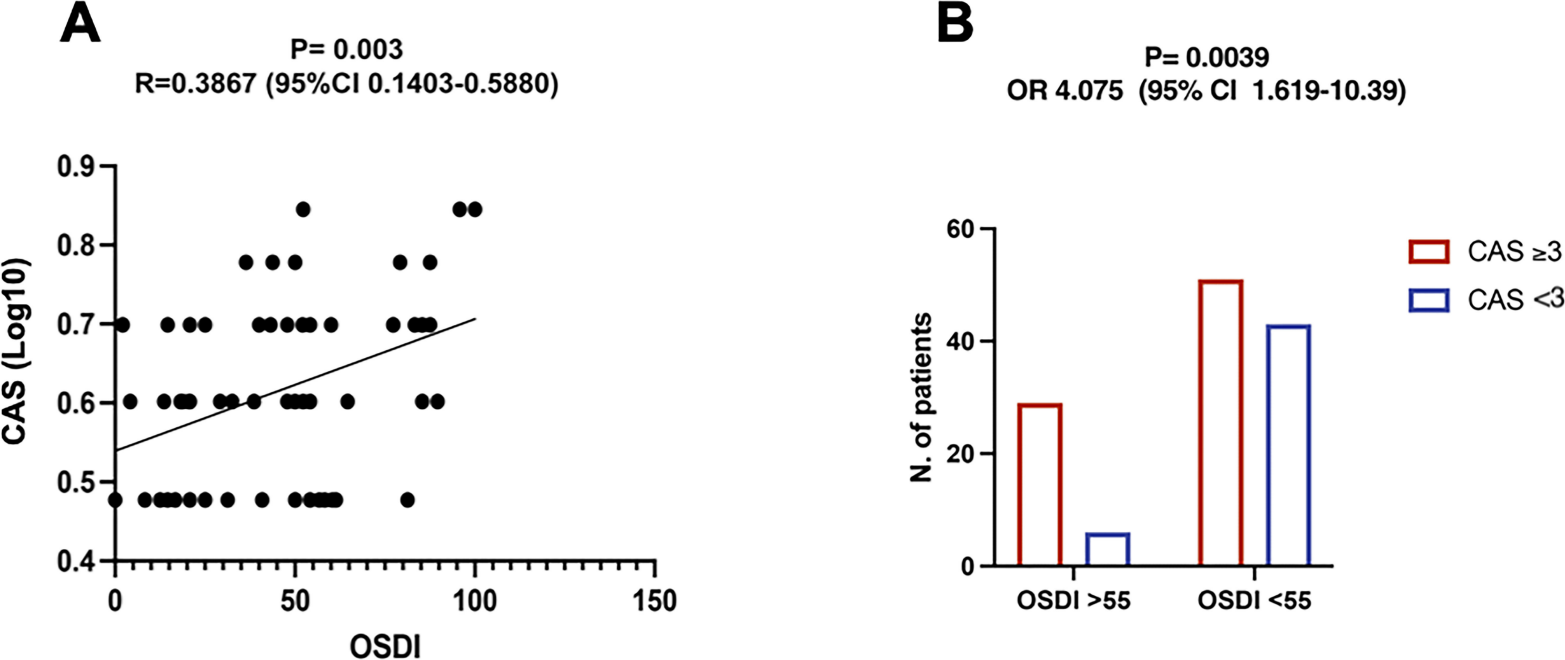
Ocular Surface Disease Index (OSDI) positively correlates with the Clinical Activity Score (CAS) in patients with moderate-to-severe active Graves Orbitopathy (GO). **(A)** Correlation between CAS (Log10 values) and OSDI within the group of moderate-to-severe active GO patients. The correlation coefficient (R) and its significance (P value) were obtained by linear regression analysis. **(B)** Comparison of the frequency of pathological OSDI between patients with CAS ≥3 and patients with CAS <3. A cutoff of 55, which was equal to the 75^th^ percentile of the study population, was used to increase specificity. P value and odds ratio (OR) were obtained by Fisher exact test.

Within patients with moderate-to-severe active GO, there was a significant direct correlation between OSDI and CAS (R= 0.3867, 95% CI 0.1403-0.5880; P=0.0030 by linear regression, using a Log10 for CAS) (**Figure 3A**). To increase specificity, we tested a cut-off value for the OSDI score of 55, which was equal to the 75^th^ percentile of the study population. Using this cut-off, the proportion of patients with pathological OSDI was significantly higher in the group of patients with CAS ≥ 3 than in patients with CAS <3 (P=0.0039; OR 4.075, 95% CI 1.619-10.39 by Fisher exact test) (**Figure 3B**). The choice for a cut-off value is arbitrary. Initially, we established a cut-off of 33, which is recognized to be diagnostic for a severe dry eye. Next, to increase the specificity, we tested a cut-off value of 55 which was equal to the 75^th^ percentile of the study population.

**Figure 4:**
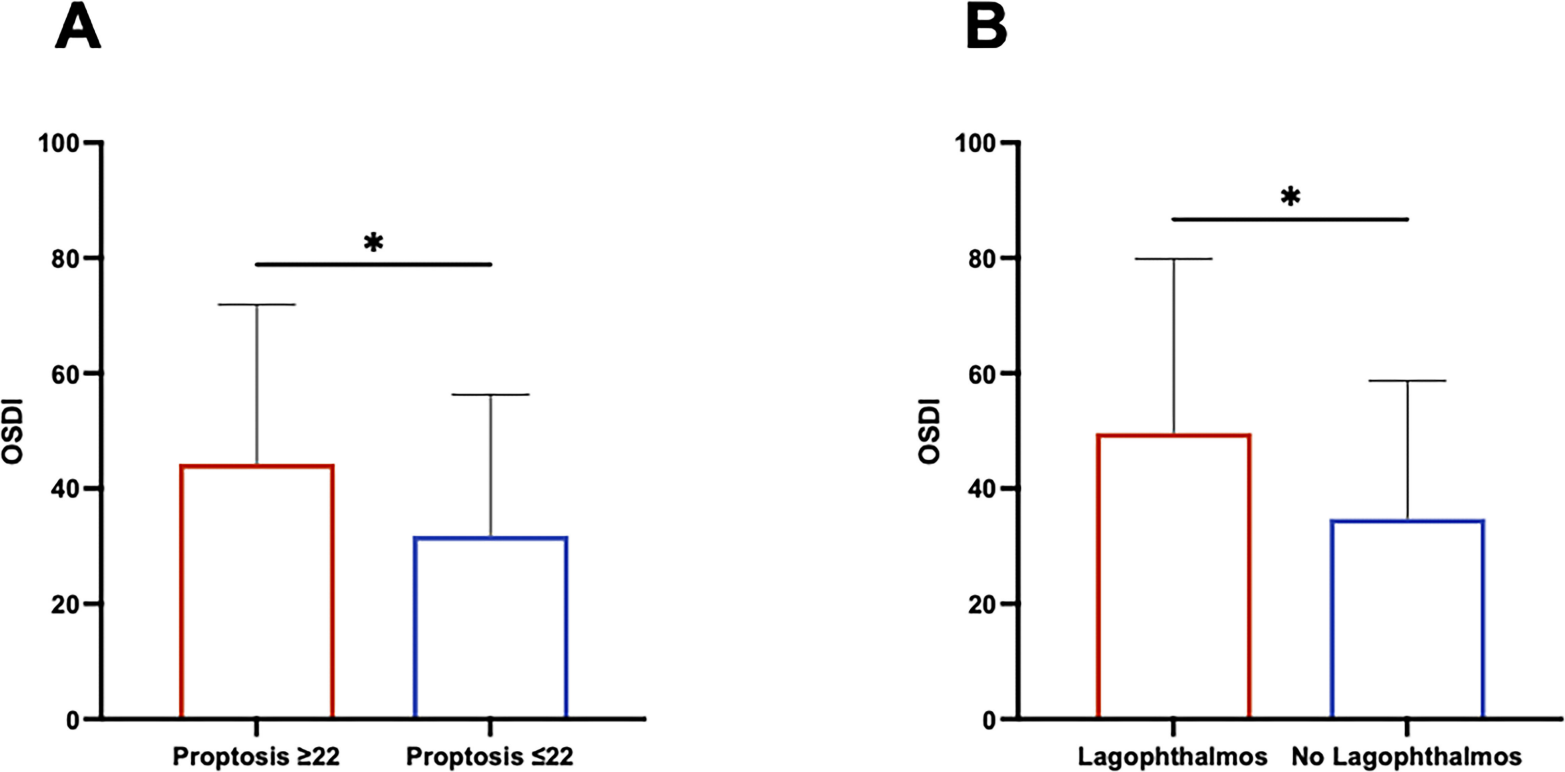
Proptosis and lagophthalmos are potential risk factors for the occurrence of ocular surface disease in patients with Graves’ Orbitopathy. **(A)** Comparison of the Ocular Surface Disease Index (OSDI) between patients with proptosis ≥22 and patients with proptosis <22. The statistical significance was assessed by Mann-Whitney test. P value significance: P <0.05, *. **(B)** Comparison of the OSDI between patients with and without lagophthalmos. The statistical significance was assessed by Mann-Whitney test. P value significance: P <0.05, *.

The correlation between OSDI and the eye features of GO was also investigated. The OSDI was significantly greater in patients with proptosis ≥ 22 mm compared to those with mild proptosis (P=0.0406 by Mann-Whitney), and in patients with lagophthalmos than in those without (P=0.0493 by Mann-Whitney), suggesting that proptosis and lagophthalmos are suitable risk factors for the occurrence of ocular surface disease (**Figure 4**). No correlation between OSDI and the other eye single features of GO, including eyelid aperture, and degree of diplopia was observed (**Figure 1**). Furthermore, OSDI did not correlate with GD and/or GO duration (**Figure 1**).

## Discussion

The OSDI is a widely used questionnaire designed to assess the symptoms and impact of ocular surface diseases, particularly DED. It was developed to provide a standardized method for evaluating the severity of DED symptoms and their effect on patients’ quality of life. Despite the well-recognized association of ocular surface disease with autoimmune thyroid diseases, particularly GO, the potential interplay between DED and the activity and severity of GO remains unexplored. Furthermore, the suitability of OSDI for assessing DED in GO patients has not been investigated. To address this gap, the present study represents the first attempt to elucidate the relationship between DED occurrence and the clinical characteristics of GO. Specifically, the investigation aimed to evaluate ocular surface health in patients with GD and moderate to severe GO, in comparison to those with GD and mild non-active GO, utilizing the OSDI score as a measure.

Our results indicate a significantly higher prevalence of ocular surface disease in patients with moderate-to-severe active GO compared to those with mild non-active disease. Consistent with previous studies, this finding underlines the impact of active inflammation and tissue remodeling on ocular surface health (6, 18–20).

The majority of mild non-active GO patients presented with OSDI <33, while OSDI scores diagnostic for ocular surface disease were more commonly observed in patients with moderate-to-severe active GO. To increase the specificity of OSDI in our study population, we also tested a diagnostic cut-off of 55. Strikingly, GO patients with CAS ≥ 3 exhibited pathological OSDI more frequently than those with CAS<3. Along with the direct correlation between CAS and OSDI, this finding suggests that inflammation plays a key role in the development of ocular surface disease in GO patients.

DED is known as a possible consequence of GO, contributing to ocular discomfort and impaired vision (11). The occurrence of this condition affects the quality of life (QoL) and daily activities, particularly among patients with additional risk factors such as advancing age, gender disparities, hormonal dysfunctions, environmental influences, and lifestyle factors (8). Our results confirm the described association between GO and DED and elucidate it further, demonstrating that the degree of GO activity significantly contributes to DED occurrence. Since GO and DED may have a serious impact on QoL, it would be interesting to investigate the association between GO-QoL and OSDI, both assessed using the appropriate questionnaire. Although we evaluated GO-QoL in this study, the retrospective design led to an imbalance in the number of QoL questionnaires collected between groups, limiting the strength of our statistical analysis. Assessing the association between QoL and OSDI scores could offer valuable insights, and future studies with a prospective design and a more balanced data collection could better explore this relationship.

The exact pathogenetic mechanisms of DED in GO remain incompletely understood, but several potential contributing factors have been suggested. Primary among these is ocular surface exposure resulting from proptosis, eyelid retraction, widening of the palpebral fissure, and lagophthalmos. Any dysfunction in eyelid function can lead to increased tear evaporation and the development of ocular surface alterations. The OSDI questionnaire, as illustrated in the 12 items detailed in **Table 2**, specifically investigates dry eye symptoms and their impact on daily activities. During the administration of the questionnaire, the focus was kept strictly on dry eye symptoms to avoid any potential confounding effects related to diplopia or other visual impairments. Although age is known to be associated with a higher prevalence of dry eye due to age-related changes in tear production and ocular surface health, we did not find a significant correlation between age and OSDI scores. One possible explanation is that the OSDI questionnaire is designed to capture the patients’ subjective experiences of dry eye symptoms in relation to their daily activities. Eyelid aperture, degree of diplopia, and visual acuity did not correlate with DED in our study.

Interestingly, we observed higher OSDI in patients with lagophthalmos and in those with proptosis ≥22, indicating that proptosis and lagophthalmos may serve as risk factors for the development of ocular surface diseases in GO patients. Consistent with previous literature (19), this finding emphasizes the correlation between GO-related orbital anatomical changes and the onset of ocular surface disease. In addition to the OSDI questionnaire, which may introduce the possibility of subjective bias, objective measures of ocular surface health, such as tear film stability or tear production tests, could provide complementary insights. Objective measurements could provide a more comprehensive understanding of how anatomical alterations can impact ocular surface health and patient QoL (21). Moreover, DED in GO seems to be predominantly of the evaporative type, with severe lower eyelid meibomian gland dysfunction (MGD) and worse lagophthalmos significantly associated with tear film instability in treatment-naive GO patients (21). This finding aligns with the importance of assessing tear film stability as part of the ocular surface evaluation in GO and supports the need for a multidimensional approach, to better address the complexity of ocular surface disease in GO patients. As the primary outcome of this study was to evaluate, as a proof-of-concept, the clinical utility of the OSDI questionnaire as a tool for exploring ocular surface health in GO patients, and given the retrospective design of our investigation, we did not include objective assessments of DED in our analysis.

The study’s cross-sectional design also limits the ability to establish causal relationships between GO activity and ocular surface disease. In addition, the lack of a pre-study sample size calculation is a limitation that needs to be considered. However, a *post hoc* analysis was performed to evaluate the power of the study. Regarding the primary outcome, the effect size calculated (Cohen’s d= 4.63) indicates a substantial difference between the groups. Specifically, the power analysis suggested that only eight patients per group would be required to achieve adequate power for this comparison. This confirms that our sample size (79 patients) was adequate. Next, we investigated the correlation between OSDI scores and CAS. The power analysis was performed by using the correlation coefficient as our effect size and setting a significance level (α) of 0.05. Although the *post-hoc* analysis suggested that a larger sample size (105 patients) would enhance the reliability of our results, the fact that we achieved statistical significance with our sample of 79 patients indicates that the correlation between CAS and OSDI is indeed detectable and meaningful in this context even in a smaller cohort.

Overall, our study demonstrates for the first time that OSDI can be used to efficiently assess the presence of ocular surface disease in GO patients and show an interesting correlation between the degree of GO activity and severity and the occurrence of DED. Our results highlight the clinical relevance of assessing ocular surface health in patients with GO, especially those with moderate-to-severe active GO. Integrating OSDI assessment with evaluation of GO severity and activity can aid in more accurate management and treatment decisions, potentially alleviating the burden of ocular surface disease and enhancing overall well-being in GO patients. Early identification and management of ocular surface disease are crucial for improving patient outcomes and minimizing ocular discomfort and visual impairment associated with GO.

Further studies, including both subjective measures like the OSDI questionnaire and objective assessments, such as tear film stability and tear production tests, are needed to confirm our results and investigate the suitability of using OSDI in clinical practice to improve the management of GO patients.

## Data Availability

The raw data supporting the conclusions of this article will be made available by the authors, without undue reservation.

## References

[1] Bartalena L Baldeschi L Boboridis K Eckstein A Kahaly GJ Marcocci C. The 2016 european thyroid association/european group on graves’ Orbitopathy guidelines for the management of graves’ Orbitopathy. Eur Thyroid J. (2016) 5:9–26. doi: 10.1159/000443828 27099835 PMC4836120

[2] Leo M Menconi F Rocchi R Latrofa F Sisti R Profilo MA. Role of the underlying thyroid disease on the phenotype of graves’ Orbitopathy in a tertiary referral center. Thyroid. (2015) 25:347–51. doi: 10.1089/thy.2014.0475 25584927

[3] Bahn RS. Current insights into the pathogenesis of graves’ Ophthalmopathy. Horm Metab Res. (2015) 47:773–8. doi: 10.1055/s-0035-1555762 26361262

[4] Bartalena L Kahaly GJ Baldeschi L Dayan CM Eckstein A Marcocci C. The 2021 European Group on Graves’ orbitopathy (EUGOGO) clinical practice guidelines for the medical management of Graves’ orbitopathy. Eur J Endocrinol. (2021) 185:G43–67. doi: 10.1530/EJE-21-0479 34297684

[5] Selter JH Gire AI Sikder S. The relationship between Graves’ ophthalmopathy and dry eye syndrome. Clin Ophthalmol. (2014) 9:57–62. doi: 10.2147/OPTH.S76583 PMC4287254 25584018

[6] Rana HS Akella SS Clabeaux CE Skurski ZP Aakalu VK. Ocular surface disease in thyroid eye disease: A narrative review. Ocul Surf. (2022) 24:67–73. doi: 10.1016/j.jtos.2022.02.001 35167950 PMC9058200

[7] Gürdal C Saraç Ö Genç İ Kırımlıoğlu H Takmaz T Can İ. Ocular surface and dry eye in graves’ Disease. Curr Eye Res. (2011) 36:8–13. doi: 10.3109/02713683.2010.526285 21174592

[8] Ismailova DS Fedorov AA Grusha YO. Ocular surface changes in thyroid eye disease. Orbit. (2013) 32:87–90. doi: 10.3109/01676830.2013.764440 23565763

[9] Gupta A Sadeghi PB Akpek EK. Occult thyroid eye disease in patients presenting with dry eye symptoms. Am J Ophthalmol. (2009) 147:919–23. doi: 10.1016/j.ajo.2008.12.007 19211095

[10] Craig JP Nichols KK Akpek EK Caffery B Dua HS Joo C-K. TFOS DEWS II definition and classification report. Ocul Surf. (2017) 15:276–83. doi: 10.1016/j.jtos.2017.05.008 28736335

[11] Perry HD Donnenfeld ED. Dry eye diagnosis and management in 2004. Curr Opin Ophthalmol. (2004) 15:299–304. doi: 10.1097/00055735-200408000-00004 15232468

[12] Schaumberg DA Nichols JJ Papas EB Tong L Uchino M Nichols KK. The International Workshop on Meibomian Gland Dysfunction: Report of the Subcommittee on the Epidemiology of, and Associated Risk Factors for, MGD. Investig Opthalmol Vis Sci. (2011) 52:1994. doi: 10.1167/iovs.10-6997e PMC3072161 21450917

[13] Nelson JD Craig JP Akpek EK Azar DT Belmonte C Bron AJ. TFOS DEWS II introduction. Ocul Surf. (2017) 15:269–75. doi: 10.1016/j.jtos.2017.05.005 28736334

[14] Gilbard JP Farris RL. OCULAR SURFACE DRYING AND TEAR FILM OSMOLARITY IN THYROID EYE DISEASE. Acta Ophthalmol (Copenh). (2009) 61:108–16. doi: 10.1111/j.1755-3768.1983.tb01401.x 6687972

[15] Rocha EM Mantelli F Nominato LF Bonini S. Hormones and dry eye syndrome: an update on what we do and don’t know. Curr Opin Ophthalmol. (2013) 24:348–55. doi: 10.1097/ICU.0b013e32836227bf 23680756

[16] Bahn RS Gorman CA. Choice of therapy and criteria for assessing treatment outcome in thyroid-associated ophthalmopathy. Endocrinol Metab Clin North Am. (1987) 16:391–407. doi: 10.1016/S0889-8529(18)30485-7 3319588

[17] Schiffman RM. Reliability and validity of the ocular surface disease index. Arch Ophthalmol. (2000) 118:615. doi: 10.1001/archopht.118.5.615 10815152

[18] Kashkouli MB Alemzadeh SA Aghaei H Pakdel F Abdolalizadeh P Ghazizadeh M. Subjective versus objective dry eye disease in patients with moderate-severe thyroid eye disease. Ocul Surf. (2018) 16:458–62. doi: 10.1016/j.jtos.2018.07.003 30297028

[19] Allam IY Lazreg S Shafik Shaheen M Doheim MF Mohammed MA. Ocular surface changes in patients with thyroid eye disease: an observational clinical study. Clin Ophthalmol. (2021) 15:2481–8. doi: 10.2147/OPTH.S317708 PMC8214558 34163131

[20] Xu N Huang D Yang H Lai Z Luo Q. Ocular surface characteristics and impression cytology in patients with active versus inactive Thyroid Eye Disease. Eye Sci. (2012) 27:64–8. doi: 10.3969/j.issn.1000-4432.2012.02.003 22678867

[21] Liao X Lai KKH Aljufairi FMAA Chen W Hu Z Wong HYM. Ocular surface changes in treatment-naive thyroid eye disease. J Clin Med. (2023) 12:3066. doi: 10.3390/jcm12093066 37176507 PMC10179143

